# Annual Address, Delivered to the Graduating Class Atlanta Medical College

**Published:** 1885-04

**Authors:** J. B. Hawthorne

**Affiliations:** Atlanta, Ga.


VOL. II. APRIL, 1885. NO. 2.

### Original Communications.

### ANNUAL ADDRESS,

DELIVERED TO THE GRADUATING CLASS OF THE ATLANTA MEDICAL COLLEGE, MARCH 2D, 1885.

BY REV. J. B. HAWTHORNE, D. D., ATLANTA, GA.

*Gentlemen of the Atlanta Medical College:*

Some years ago, in a Southern town, a convention of public-spirited men assembled in the interest of education. The man selected to make the opening address was an illiterate farmer. He began his speech by saying that he had accepted the place because he believed that an exhibition of his ignorance would serve to emphasize the importance of a liberal education. If the committee entrusted with the responsibility of choosing an orator for this occasion aimed to help the cause of medical science by a similar exhibition, they deserve to be congratulated upon the wisdom of their choice. My knowledge of materia medica does not extend very far beyond "Perry Davis' Pain Killer," "St. Jacob's Oil" and "Mrs. Winslow's Soothing Syrup." Earlier in life I had a passion for the healing art. I did a considerable practice among the negroes of my father's plantation, the results of which have never been published in any medical journal. My leading remedy was bread pills, and with it I made many a signal cure. But for one serious mistake I might have continued in the same business to this day. Eager to give relief in a desperate case of cramp colic, I administered an over dose of "Number Six." It cured the disease, but almost killed the patient. Since then I have left doctoring to the doctors.

I have learned very little about medicine, but a great deal about medicine-men. Not much do I know of law, but I have been a very diligent and critical observer of lawyers. I have never made a study of politics, but in the knowledge of politicians I claim to be profound.

I desire to state briefly a few things concerning professional men and then offer a little advice to these young gentlemen who are about to begin a professional career.

1. There is a vast amount of mediocrity in every profession which passes for superiority. It is not difficult for a man to deceive the multitude, and to make them believe that he possesses extraordinary ability. I have seen physicians with scarcely any real merit who had more business than they could attend to, and in the same community I have seen other physicians of culture and skill starving for the lack of patients. In the ranks of every profession, men are canonized for superior wisdom who are the merest tyros in comparison with others whose names are seldom spoken.

The explanation of this fact is not difficult. Some men carry themselves more wisely than others. They have a portlier and statelier demeanor. A man who knows his weakness and emptiness learns how to appear strong and full, and to move among men with a commanding air, while a man of genuine merit is simple and unpretending. A young man, whom I had taught the elements of arithmetic and English grammar, went to a medical college a few months where he obtained a diploma. He had an estate worth four or five thousand dollars, which he converted into money, and then removed to New York city.

He rented a magnificently furnished office, near Fifth Avenue, bought a pair of horses and an elegant carriage, and then offered his professional services to the public. I saw him a few years afterwards, and he had the bearing of a man carrying all the learning of a half dozen universities in his head. He winked at me very significantly, and told me that he was making ten thousand a year, and would soon marry the only child of a merchant prince.

The doctor who has the genius of taciturnity, whose utterances are few and carefully premeditated, and who in the midst of excitement knows how to keep cool and look wise, will grow rich where an unpretentious man of superior ability would starve. Anybody can pass for a wise man by simply concealing his ignorance. The president who never makes a speech, but responds to the calls of the admiring multitude with a simple bow of his august head, is supposed to know more than Thomas Jefferson. The doctor who never commits himself to anything less certain than a truism, who understands the diplomacy of gesture, and knows how to wrap himself in a robe of mystery, is supposed to hold within his cranium a vast reservoir of knowledge. His silence means profundity. When men discuss knotty questions in his presence he lifts his eyebrows and smiles, as if he was about to say : "How easily I could set the babblers right."

Bulwer says: "The more a man desirous of passing for more than his real worth can contrast by a dignified silence the garrulity of trivial minds, the more the world will give him credit for what he does not possess." When we see a strong box, with its lids held down by iron clasps, and secured by a jealous padlock, we at once suppose it to contain articles of great value. We could hardly believe one sincere who should tell us that the box contained only scraps—the odds and ends of things—which would be just as safe wrapped in a cheap newspaper. The generality of people imagine fathomless depths of wisdom in a man who is seldom heard to speak. And when such a man does speak they feel that they are listening to an oracle whose utterances are infallible.

Silence means mystery, and we never take liberties with mysterious men. The children never run to the door to greet the silent doctor. The mother never invites him to remain to tea. Such greatness must be viewed and enjoyed at a distance.

There is no profession that accommodates what I have named "the genius of taciturnity" so well as that of medicine. There is no law which requires a physician to explain to the patient the nature and cause of his disease. There is none which makes it his duty to expound the philosophy of his treatment. He can produce a profound impression by a few words of "learned length and thundering sound." He can invest a common poultice with marvelous virtue by calling it an "emollient cataplasm." He can quiet the fears of the agitated family by telling them that he is proceeding *secundum artem.* If he leaves the chamber of sickness without imparting any information, either to the patient or his family, he is regarded as much superior to the man who gives a clear and full explanation of the disease and of the treatment adopted.

Young gentlemen, allow me to express the wish that you may never condescend to such trickery in the practice of your noble profession. Never attempt to pass for more than you are worth. Let the world know the results of your study and experience. Give to those whom you serve a reason for the faith that is in you. Aim to be transparent as the day. Do not try to be old while you are young. Tarry in Jericho till your beard is well grown. Do not force nature into any premature developments. Build solidly and honestly. Then your success will be real. The esteem and confidence of men will be doubly sweet to you, because they have been fairly won. Conscious of the rectitude of your course, your labor will not only be profitable to others, but a constant source of delight to yourselves.

2. The majority of young doctors are timid and shy. The same is true of most men in beginning the practice of any profession. The first time I was called Reverend I almost fainted. I was not ashamed of my calling, but ashamed of myself; ashamed of my unfitness for it; ashamed of my ignorance and lack of experience. If at the conclusion of my first sermon any member of my congregation had pronounced me a heretic, I think that I would have pleaded guilty, and besought his forgiveness.

A young minister chose for the subject of his maiden sermon "The knowledge of God." Profoundly conscious of his weakness, and surrounded by a group of learned theologians, he felt the importance of weighing his words. With fear and trembling he announced as his first proposition, "God knows all things." Just then one of the learned clergy drew a long breath, and the young man, apprehensive that he had made an extravagant assertion, added, "That is to say, my beloved brethren and sisters, though my acquaintance with the subject is exceedingly limited, I venture in great weakness and much confusion to express the humble and feeble opinion that there are not *many* things which God does not know."

That was timidity resulting from inexperience and insufficient preparation for his work.

These young gentlemen who have received their diplomas this evening will walk the streets to-morrow with a very nervous step. When some one says, "How are you, doctor?" they will look very shy, not knowing whether it is intended for a courtesy or a joke.

Everybody feels free to joke beginners in any profession. A wag said to a young physician: "Doctor, are you getting any practice?" "Very little; I sit here day after day like Patience on a monument." "By and by," replied the wag, "it will be worse than that. You won't have Patience on a monument there, but you will have monuments on your patients."

A young surgeon in the West was called to see a man who had received fatal injuries in a railroad disaster. The town paper, in describing the accident, said: "Dr. Jones was called in and under his prompt and skillful treatment, the poor man died on Wednesday night."

Young gentlemen, do not allow yourselves to be discouraged by such experiences. If you are true to yourselves, a few years hence, you will be beyond the reach of the fun-makers, and the men who now joke you will treat you with the most dignified courtesy.

I like to see a young man sensitive, nervous, tremulous and shy in undertaking a work that is noble and great. It is a good sign. It indicates earnestness and a lofty ideal. It is not the scrub-horse, bnt the thoroughbred, that is nervous and restive. The sight of the race-course, and of his competitors in the approaching struggle, makes him tremble and prance with excitement. The men who run best in a professional career are the men of mettle—the sensitive, nervous, excitable men.

3. In any profession there are only a few men trying to turn up new earth; to make some original contribution to the cause which they serve. The majority would rather inherit than create. They are loyal to precedent. They never question recognized authority. They withdraw the hand of fellowship from a brother as soon as he begins to think for himself. They believe and practice only what they have been taught, and are expected to believe and practice.

The true knight of a profession is he who dares to question the infallibility of the teachers and the standards; who, with the hemlock or the cross in sight, would expose an error, or announce what he has fairly and honestly found to be a better theory or a wiser practice. The naked tree of winter lifting up its dead arms to the sky, is not more ennobled when, flooded with summer sap, it climbs to a soul in flower and fruit, than a man when he resolves to think for himself, and to put forth as fruit his own faith, his own thought, and his own life.

It is the wisdom of God which says: "Prove *all* things; hold fast that which is good." I believe in dynamite; not that sort which Fenian and Socialistic deviltry employs to blow up bridges, palaces and Parliament buildings, but the intellectual and moral dynamite which blows into fragments and out of sight established error and wrong. I believe in assassination, not of men, but of false principles and false customs. In my heart of hearts I bless the incendiary who dares to apply the torch of truth to a canonized falsehood.

The worshipers of the veiled Isis affirm that the chosen incense of their goddess is scentless to all but her TRUE worshipers. Only the morally brave—the men who are ever ready for the altar of sacrifice—know the beauty and preciousness and glory of truth.

Young gentlemen, if you have made up your minds to be the slaves of precedent and custom and routine; if you have determined that you will not hazard your bread and butter, your prospects of material gain, by any sort of independent thinking and acting; if you have decided to be simply maggots among maggots, each striving to be only a little larger and better fed than the rest, then I not only pity you and the medical profession, but I pity the unfortunate people upon whom you are about to be turned loose.

Do not imagine that I would encourage arrogance and impudence and reckless audacity. The young physician ought to be brave and progressive, but he should be very sure that he has discovered a better way, before he sets aside the wisdom of his seniors. Human life is something too precious to be sacrificed to self-conceit, or the mere love of experiment.

An old physician sent a young doctor who had never treated a case to attend one of his patients, a country woman of not much education, but with a good stock of common sense. The young man went to the sick room, and proceeded to examine the case. The patient objected. "Why do you object?" said the doctor. "Bekase, sir, you come here just to larn, and I'll die 'fore you shall larn on me."

4. In every profession, and especially in that of medicine, there is much of what is called *one-sidedness*. There are men who develop only in a single direction. There are lawyers who are nothing but lawyers—walking digests of the laws that pertain to deeds, demurrers and hereditaments. There are doctors who are nothing but doctors. Their names and faces suggest nothing but pills and plasters and blisters. There are clergymen who are nothing but clergymen. They dress like clergymen, talk like clergymen, walk like clergymen, smile like clergymen, and eat like clergymen. The parsonic qualities are conspicuous on every occasion, justifying the remark of Dean Swift,—"The world is divided into three classes, men, women, and preachers."

Mrs. Sommerville wrote a charming book on "The Connection of the Sciences." But it is not only the sciences that have a family kinship. Every faculty of the human intellect and soul is akin to every other faculty, and is in some degree dependent upon it. You cannot enrich one department of the human mind without improving at the same time and by the same means every other department.

It may be true that the poet is born, not made, and yet we know that the world's greatest poets were distinguished for the variety of their acquisitions. Virgil and Shakespeare and Milton and Scott were men of vast and varied information and culture. There is no kind of knowledge that may not be serviceable to a man in any calling that he may enter. All kinds of knowledge may be made to blend with and enrich the one kind to which we attach our reputation. The lawyer may get from the study of the classics a certain mental development and equipment which he would never have if he should confine himself to books of law. When the statesman adds to his knowledge of government an acquaintance with philosophy, science and history, art and religion, he doubles his capacity for the special work in which he is engaged. A minister of the gospel may know the Bible from beginning to end, but if he is familiar with no other book he is very imperfectly equipped for the duties of his sacred vocation. The physician needs for his work a very high degree of intellectual development, but he will never have it if he limits his intellectual labor to matters connected directly with his profession.

Young gentlemen, this world is a great school-house, and we are in it, not for the education of a single faculty, but for the training of the whole man. Our capabilities are complex, and in building character we should build up on all sides, that we may have variety and amplitude of being.

We should build with reference to the life to come. A man may become a great artist, but when he enters eternity it will not be as an artist, but as a man. When death comes you will be no longer physicians. Beyond the grave there is no sickness to be cured. When you stand before God in the judgment of the great day it will not be as physicians but as men.

"We are not fragments; we are wholes. We are not types of single qualities; we are realities of mixed, various, countless elements." No one is placed in this world to improve just one talent, but to develop, enrich and utilize his entire being.

Let us strive for breadth of character and culture. Let our thoughts and sympathies sweep over a vast field. Let the commerce of our minds and hearts extend to distant shores. The small retail dealer trades only with the neighborhood, but the merchant prince touches the four quarters of the globe.

I want to know my physician not merely as an individual who can set a bone and write a prescription. I want an acquaintance that extends beyond his professional character. I want to know his mind, and heart and soul. In other words, I want to know him as a *man*. If he is only qualified to minister to my poor body, he is not the physician that I need. If he cannot stimulate my mind and comfort my heart and sympathize with the aspirations that leap like angels from the temple of my soul, then give me one who can.

5. It is a lamentable fact that many physicians are materialists. They do not recognize the dual nature of man. They do not see that he is both material and immaterial—body and spirit—mortal and immortal. They view him simply as an animal, whose prospects and existence are bounded by the grave. Regarding him merely as an animal, they have no reverence for his nature. They drench him and cut him and saw him simply for their own edification and entertainment.

*Man is an animal.* It is true that he has a thumb to his hand, where other animals have fingers only, and that his skull sits on the top of the vertebral column; but these only show that he is a higher variety of animal. There is not a joint, or a fibre, or a sense, or a function of blood and nerve in man which is not in the quadruped also. Men are fond of dress and music. They dance and sing, and love and hate. They build and sow and plant and weave. All this is true of the lower animals. Nor does the resemblance stop here. Human society often presents the appearance of a menagerie of animals—some proud and some humble, some timid and shy, and some bold and fierce, some playful, and many stupid. There are two-footed calves and mules and hyenas. In every congress, in every parliament, in every convention, and in every fashionable assembly, you may hear the bray of donkeys, and the hissing of geese and serpents.

But when we have said that man is an animal, we have expressed only a fragment of the truth concerning his being.

If there is a science which says that man is another link in the animal chain, there is a higher science which says that in him the link fastens on to angels, thrones and dominions.

The Greek word for man was formed of three other words, signifying, *to turn the eye upwards.* Man's eye overleaps the foregrounds of life, which limit the vision of animal sense, and sees beyond the horizon of this material realm, a building of God, a house not made with hands, eternal in the heavens.

Man is made but little lower than the angels. Man is God's temple. Man developed in his higher nature is God's image. Man is the centre of the physical universe. Before him nature fades as a frame before the portrait it surrounds.

Sinai is but a hillock when Moses stands upon it. Thermopylæ is nothing when Leonidas is there. The seven hills of Rome are but ant-heaps when Cicero's voice is heard. Man is greater than the starry heights to which he lifts his longing eyes, for the day cometh when he shall put them all beneath his feet. When the King of kings and Lord of lords entered our world and dwelt among us, it was in the person of a man.

Young gentlemen, to the extent that you repudiate this conception of man you will prove yourselves unworthy of your high vocation. Without that reverence for man which is born of faith in God's Word, you will have no deep sense of responsibility. You will lack that delicate and helpful sympathy which a human sufferer is so quick to detect. You will degenerate into mere hirelings, whose only incentive to labor is material reward.

